# Developing a Robotic Surgical Platform Is Beneficial to the Implementation of the ERAS Program for Colorectal Surgery: An Outcome and Learning Curve Analysis

**DOI:** 10.3390/jcm12072661

**Published:** 2023-04-03

**Authors:** Chun-Yen Hung, Chun-Yu Lin, Ming-Cheng Chen, Teng-Yi Chiu, Tzu-Wei Chiang, Feng-Fan Chiang

**Affiliations:** 1 Division of Colorectal Surgery, Department of Surgery, Taichung Veterans General Hospital, 1650 Taiwan Boulevard Sect. 4, Taichung 40705, Taiwan; 2 Department of Food and Nutrition, Providence University, Taichung 43301, Taiwan

**Keywords:** enhanced recovery after surgery, ERAS, colorectal surgery, minimally-invasive, laparoscopic, robotic, learning curve, outcome, compliance

## Abstract

Background: Robotic surgery and ERAS protocol care are both prominent developments and have each become global trends. However, the effects and learning curves of combining robotic surgery and ERAS care in colorectal resection have not yet been well validated. This study aimed to present our real-world experience and establish the learning curves necessary for the implementation of an ERAS program in minimally-invasive surgery for colorectal resection, while also evaluating the impact that the development of the robotic technique has on ERAS outcomes. Methods: A total of 155 patients who received elective, minimally-invasive surgery, including laparoscopic and robotic surgery for colorectal resection, with ERAS care during the period June 2019 to September 2021 were included in this retrospective analysis. Patients were divided chronologically into five groups (31 cases per quintile). Patient demographics, tumor characteristics, perioperative data, ERAS compliance, and surgical outcomes were all compared among the quintiles. Learning curves were evaluated based on ERAS compliance and optimal recovery, which are composed of an absence of major complications, postoperative length of stay (LOS) of no more than five days, and no readmission within 30 days. A multivariable logistic regression model was used to assess factors associated with postoperative LOS. Results: There were no statistically significant differences seen overall or between the quintile groups in regards to demographic and tumor characteristic parameters. A total of 79 patients (51%) received robotic surgery, with the ratio of robotic groups rising chronologically from zero in the first quintile to 90.3% in the fifth quintile (*p* < 0.001). The median compliance rate of total ERAS protocol was 83.3% overall, 72.2% in the first quintile and 83.3% in the 2nd–5th quintiles (*p* < 0.001). A total of 85 patients underwent optimal recovery after surgery, four patients in the first quintile, 11 patients in the second quintile, and 21, 24, 25 patients in the 3rd–5th quintiles respectively (*p* < 0.001). There were significant improvements from early to later groups upon postoperative LOS (*p* < 0.001). In addition, the surgical outcomes including first oral intake within 24 hours after surgery, time to first stool and early termination of intravenous fluid administration showed significant improvement among the quintiles. A multivariable logistic regression model demonstrated that robotic surgery was superior to laparoscopic surgery upon postoperative LOS (odds ratio = 5.029, 95% confidence interval [CI] = 1.321 to 19.142; *p* = 0.018). Conclusions: Our experience demonstrated that an effective implementation of the ERAS program in minimally-invasive colorectal surgery requires 31 patients to accomplish the higher compliance and requires more cases to reach the maturation phase for optimal recovery. We believe that developing a robotic platform would have no impact on the learning curve of ERAS implementation. Moreover, there is a beneficial effect on the postoperative length of surgery provided through the combination of ERAS care and robotic surgery for patients undergoing colorectal resection.

## 1. Introduction

Enhanced recovery after surgery (ERAS) protocols are multimodal perioperative care pathways designed to achieve early recovery for patients after surgical procedures through maintaining preoperative organ function and reducing any profound stress response following surgery. In the past two decades, ERAS protocol has been widely accepted worldwide and has been proved effective in bringing about shorter lengths of hospital stays, decreased postoperative pain and need for analgesia, decreased complications and readmission rates, and increased patient satisfaction. However, the effective implementation of ERAS requires close multidisciplinary teamwork and learning curves in order to best adjust the protocols into daily practice.

In Taiwan, promotion of the ERAS program has exploded over the past three years. The *Taiwan Chapter, ERAS Society* was established in July 2019, with more and more medical providers developing their tailored ERAS protocols on a diverse array of surgical procedures. In our institution, the implementation of ERAS programs for colorectal surgery patients began in June 2019. It is worth noting that a growing trend in the adoption of robotic surgery as a minimally-invasive technique for colorectal surgery has been happening since 2020 in our institution, while we have also carried out ERAS protocols for robotic surgery during its early stages of development. 

The primary objective of this retrospective study was to present our real-world experience and establish the learning curves necessary for the implementation of an ERAS program for patients undergoing minimally-invasive surgery for colorectal resection, while also evaluating the impact that the development of robotic surgery has on the outcomes of ERAS.

## 2. Materials and Method

We collected 176 adult patients who had received elective, minimally-invasive surgery, including laparoscopic and robotic surgery for colorectal resection with ERAS care during the period June 2019 to September 2021. Nineteen patients required conversion to open surgery due to severe adhesion or anatomic difficulty, while two patients required immediate postoperative intensive care due to unstable vital signs during surgery, and in turn were all excluded. Overall, a total of 155 patients were ultimately included in the retrospective analysis. 

All patients had received the same ERAS programs and equivalent forms of treatment from the same multidisciplinary team. Our ERAS protocol was revised according to the ERAS Society Guidelines [1] and consisted of 18 core elements, including 4 preadmission items, 4 preoperative items, 3 intraoperative items and 7 postoperative items, as summarized in Table 1. 

Patients were divided into 5 groups chronologically (31 cases per quintile). Patient demographics, perioperative data, tumor characteristics, surgical outcomes, and ERAS compliance were all compared among the quintiles. Learning curves of effective ERAS implementation were evaluated based on ERAS compliance and optimal recovery, which are composed of an absence of major complications, postoperative LOS of no more than 5 days, and no readmission within 30 days. We compared the outcomes between the laparoscopic and robotic groups and carried out multivariable logistic regression analysis to figure out significant direct correlations.

All statistical analyses were performed using PASW Statistics software (SPSS version 22.0). Continuous variables were expressed as mean (SD) or median (Q1–Q3) and were compared among groups using either one-way analysis of variance (ANOVA) or Kruskal–Wallis test or Mann–Whitney U test. Categorical data were expressed as numbers (percentage) and were compared using either Pearson’s Chi-square test or Fisher’s exact probability test or Yates’ Correction for Continuity. A *p*-value of <0.05 was considered statistically significant. This retrospective study was approved by the Institutional Review Board of Taichung Veterans General Hospital (No: CE21319A) and written informed consent was obtained from each patient.

## 3. Results

### 3.1. Patients’ Demographics and Tumor Characteristics

Overall, 155 patients were included in this analysis (Table 2). Among these patients, 85 (54.8%) were male, the mean age was 61.3 years, and the mean BMI was 24.9 kg/m^2^, with 117 of the patients (75.5%) having an ASA ≤ 2, and 35 patients (22.6%) citing previous cigarette use. There were 94 patients (60.6%) diagnosed with any type of comorbidity, including hypertension (45.2%), diabetes (25.2%), and cardiovascular events (9.7%), with two or more having primary cancer (4.5%). Median preoperative hemoglobin was 13.1 gm/dL. There were 32 patients (20.6%) who had undergone previous abdominopelvic surgery, and 22 (14.2%) with neoadjuvant CCRT. Among all the above demographic parameters, there were no statistically significant differences seen between the overall patients and each quintile group. In total, 29 patients (18.7%) experienced a right-sided colon tumor, 65 (41.9%) had a left-sided colon tumor and 61 (39.4%) had a rectal tumor. There were 136 patients (87.7%) having malignant tumors, of whom 16 experienced a T4 lesion (10.3%). 

The tumor characteristics, including tumor location, malignancy, tumor stage, safe margin, and residual tumor after neoadjuvant CCRT, among the quintile groups, were all comparable (Table 3). Group 5 showed a higher percentage of rectal tumors than Group 1, which may be explained due to the use of robotic surgery on a higher percentage of patients in Group 5 than in Group 1.

### 3.2. Compliance of ERAS Program

The median compliance rate for total ERAS protocol was 83.3% overall, 72.2% in Group 1, and 83.3% in Groups 2, 3, 4 and 5 (Table 4). In subgroup analysis, the median compliance rate for postoperative items was only 71.4%, while other items were 100%. The compliance with the ERAS program between the quintile groups demonstrated a statistically significant difference in total protocol, intraoperative, and postoperative items. The overall compliance rate for individual ERAS elements is shown in Supplementary Figure S1. 

### 3.3. Perioperative Data and Clinical Outcome Analysis

Perioperative data and clinical outcome analysis of the patient groups are listed in Table 5. In total, we included 76 patients (49%) in the laparoscopic group and 79 patients (51%) in the robotic group. It is worth noting that the ratio of robotic groups rises chronologically, with zero being seen in Group 1, 42% in Group 2, 48.4% in Group 3, and 74.2% and 90.3% in Groups 4 and 5, respectively (*p* < 0.001). Mean operative time overall was 250.3 minutes, with the later groups having a relatively longer time than the earlier groups, but with no statistical significance. A total of 85 patients underwent optimal recovery after surgery, with four patients in Group 1, 11 patients in Group 2, and 21, 24, 25 patients in Group 3 to Group 5 respectively (*p* < 0.001). The median length of stay after surgery was 5 days overall, 8 days in Group 1, 7 days in the Group 2, 5 days in Group 3, and 4 days in the Group 4 and 5 (*p* < 0.001). Both absence of major complications and readmission within 30 days showed no statistically significant differences among the quintile group. Bowel functional outcomes including first oral intake within 24 hours after surgery (*p* = 0.001), time to first stool (*p* < 0.001), and early termination of intravenous fluid administration by POD-3 (*p* < 0.001) showed significant advancement among the quintiles.

### 3.4. Comparison of Laparoscopic Surgery and Robotic Surgery

**Figure 1:**
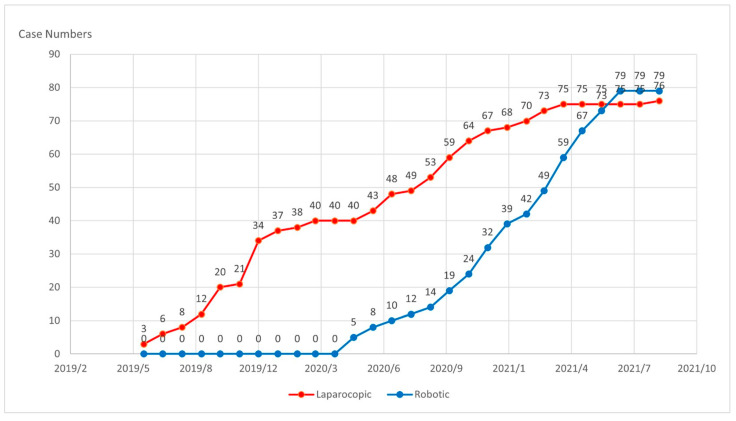
Cumulative numbers between Laparoscopic and Robotic group.

The first case in the laparoscopic group and that in the robotic group were separated by a period of 12 months. The case numbers in the robotic group equaled those in the laparoscopic group during the period of 14 months (Figure 1). The median compliance of total ERAS protocol was 88.9% in the robotic group and 83.3% in the laparoscopic group (*p* < 0.001). The median postoperative LOS was 4.1 days in the robotic group and 6 days in the laparoscopic group (*p* = 0.016) (Table 6). A multivariable logistic regression model demonstrated that robotics was superior to laparoscopic surgery upon postoperative LOS with statistically significant differences after adjustment for confounding variables (odds ratio = 5.029, 95% confidence interval [CI] = 1.321 to 19.142; *p* = 0.018) (Table 7).

## 4. Discussion

Many experts have indicated that rather than sticking to complete adherence to ERAS protocols, implementing a well-tailored ERAS protocol makes it easier to apply to clinical practice, while also offering equivalent benefits [2,3]. Five basic elements of care, including preoperative patient information, multimodal analgesia, avoidance of fluid overload and hypovolemia, no nasogastric tube and early oral feeding, as well as early mobilization, were demonstrated a long time ago to be the key components for ERAS in colonic surgery [4]. These five elements were also enough to secure enhanced recovery and a length of stay within 2 to 4 days after open colonic surgery. Toh et al. [5] initiated a questionnaire survey involving 300 colorectal surgeons in Australia and New Zealand learning that eight interventions, including preoperative anemia correction, minimally-invasive surgery, early in-dwelling catheter removal, preoperative smoking cessation, preoperative counselling, avoidance of drains in colon surgery, avoiding nasogastric tubes and early drain removal in rectal surgery were all considered relatively important elements towards improving ERAS programs. Our ERAS protocol adopted five key components and other generally acknowledged elements of care. On top of that, our ERAS protocol was implemented by a group of fixed, well-trained members, including surgeons, anesthesiologists, nurse practitioners (NPs), nutritionists and case managers.

We included a total of 155 patients and used the data-splitting method [6] by placing the patients into five consecutive groups of 31 each in order to better evaluate our learning curves with regards to ERAS care. A prospective review of 380 patients who underwent elective open colorectal surgery under ERAS protocol from the period 2011–2017 in a single institution indicated that a minimum of 76 patients are required in order to achieve a significantly higher rate of ERAS compliance and optimal recovery [7]. Michal, et al. concluded that introducing the ERAS protocol is a gradual process and its compliance at the level of 80% or more requires at least 30 patients and a period of approximately 6 months [8]. Having at least 30 cases is required for Phase I of the learning curve and has been a consensus for robotic colorectal surgery [9]. After analyzing previous studies, a cutting-off point at 31 cases for our study was deemed reasonable. 

The compliance of ERAS protocol still plays a critical role in improving short-term outcomes [10]. A multicenter, prospective cohort study involving 2084 consecutive adult patients who had undergone elective colorectal surgery in Spain demonstrated that an increase in ERAS adherence appeared to be associated with a decrease in postoperative complications (OR, 0.33; 95% CI, 0.26–0.43; *p* < 0.001) [11]. Wei et al. [12] confirmed that an increase in ERAS protocol adherence was associated with a further decrease in hospital length of stay. The latest ERCOLE study included prospective data from 1,138 patients who had undergone minimally-invasive colorectal cancer surgery in Italy and has shown that adherence to the ERAS program of up to 75% could be considered satisfactory in order to reach the goal of functional recovery [13].

In our experience, the median compliance regarding total ERAS protocol among all groups was 83.3%, and it underwent significant progress from 72.2% in Group 1 to 83.3% in Group 2 while staying relatively constant from Group 3 to Group 5 (*p* < 0.001). In subgroup analysis, we discovered a similar trend in median compliance regarding intraoperative and postoperative ERAS items (*p* < 0.001). In terms of clinical outcomes, the optimal recovery showed an increasing trend in the 2nd quintile and started to plateau after the third quintile. Postoperative LOS and bowel functional outcomes (early oral intake within 24 h, time to first stool and early termination of intravenous fluid administration by POD-3) had the similar trends with statistically significant differences between the quintile groups. Comprehensively, based on these finding we concluded that an effective implementation of the ERAS program in minimally-invasive colorectal surgery required 31 patients to accomplish the higher compliance and required more patients to reach the maturation phase for optimal recovery. 

In general, the adherence of preadmission and preoperative items should be independent from the surgical technique adopted (open, laparoscopic, robotic methods). We speculated that robotic surgery might have benefits on adherence of intraoperative and postoperative items because a robotic surgical platform with proficient technique helps surgeons achieve a more precise dissecting tissue plane and causes less bleeding damage. For instance, this could convince surgeons of the resultant security caused by the avoidance of intra-abdominal or pelvic drain. In this study, robotic surgery was correlated with higher compliance with total ERAS protocol, intraoperative, and postoperative items in univariate analysis. However, the multivariate comparison demonstrated ERAS compliance significantly correlated to the sequence of quintile groups, rather than the robotic surgery (Supplementary Table S1). On the other hand, the fact that robotic surgery was superior to laparoscopic surgery upon postoperative LOS and statistical significance was demonstrated after adjustment for confounding factors. Consequently, we made a conservative inference that developing a robotic platform would have no impact on the learning curve of ERAS implementation and there is a beneficial effect on the postoperative length of surgery provided through the combination of ERAS care and robotic surgery for patients undergoing colorectal resection.

Depending upon the future advancements made in robotic surgery, medical personnel will be able to easily perform more complex surgical procedures such as intersphincteric resection (ISR), pelvic autonomic nerve preservation and multivisceral resections [14,15,16]. However, these complex operations would affect a physician’s perspective of ERAS implementation as they are different from conventional laparoscopic surgery, for instance, the duration of a urinary catheter or drainage tube placement. The consequence of this limitation is that we should categorize the various types of procedures seen between laparoscopic and robotic surgery as more cases are taken on, for required study.

## 5. Conclusions

Robotic surgery and ERAS protocol care have each undergone their own prominent development and in turn become global trends, with rapid progress being seen in both safety and efficacy when compared to conventional surgery over the past decade. We believe that implementing a combination of the robotic surgery and ERAS care will result in a highly promising method for use in future colorectal surgeries.

## Figures and Tables

**Table 1:** Tailored ERAS protocol.

Preadmission	Dedicated preoperative counselling
	2. Cessation of smoking
	3. Screening and treatment of anemia before surgery
	4. Nutritional assessment and support as needed
Preoperative	5. Prevention of nausea and vomiting
	6. Avoidance of routine sedative medication
	7. Antimicrobial prophylaxis and skin preparation
	8. Preoperative fasting and carbohydrate treatment
Intraoperative	9. Standard anesthetic protocol
	10. Prevention of intraoperative hypothermia
	11. Avoidance of intra-abdominal or pelvic drain
Postoperative	12. Avoidance of nasogastric intubation
	13. Multimodal postoperative analgesia
	14. Near-zero fluid balance therapy
	15. Early oral intake within 24 h after surgery
	16. Termination of intravenous fluid administration by *POD 3
	17. Removal of urinary catheter by *POD 3
	18. Early mobilization by *POD 3

*POD = Postoperative Day.

**Table 2:** Demographic analysis of patient groups.

Parameters	Overall	Group 1	Group 2	Group 3	Group 4	Group 5	*p* Value
Number of patients, *n*	155	31	31	31	31	31	
Males, *n* (%)	85 (54.8%)	22 (71%)	17 (54.8%)	17 (54.8%)	16 (51.6%)	13 (41.9%)	0.242 ^P^
Mean age, years ± SD	61.3 ± 14.2	61.4 ± 13.6	64.4 ± 16.0	63.8 ± 13.4	60.4 ± 12.8	56.4 ± 14.3	0.176 ^A^
BMI, kg/m^2^ ± SD	24.9 ± 4.2	25.3 ± 4.0	24.4 ± 5.1	25.3 ± 3.6	24.4 ± 3.2	24.9 ± 4.9	0.577 ^K^
ASA ≤ 2, *n* (%)	117 (75.5%)	26 (83.9%)	23 (74.2%)	24 (77.4%)	23 (74.2%)	21 (67.7%)	0.681 ^P^
Cigarette use, *n* (%)	35 (22.6%)	12 (38.7%)	5 (16.1%)	6 (19.4%)	7 (22.6%)	5 (16.1%)	0.180 ^P^
Any comorbidity, *n* (%)	94 (60.6%)	19 (61.3%)	19 (61.3%)	22 (71.0%)	18 (58.1%)	16 (51.6%)	0.637 ^P^
Hypertension, *n* (%)	70 (45.2%)	13 (41.9%)	15 (48.4%)	16 (51.6%)	14 (45.2%)	12 (38.7%)	0.861 ^P^
Diabetes, *n* (%)	39 (25.2%)	7 (22.6%)	5 (16.1%)	9 (29.0%)	9 (29.0%)	9 (29.0%)	0.700 ^P^
Cardiovascular, *n* (%)	15 (9.7%)	1 (3.2%)	4 (12.9%)	6 (19.4%)	1 (3.2%)	3 (9.7%)	0.156 ^P^
Multiple primary cancers, *n* (%)	7 (4.5%)	3 (9.7%)	1 (3.2%)	1 (3.2%)	1 (3.2%)	1 (3.2%)	0.664 ^P^
Median preop hemoglobin, gm/dl (IQR)	13.1 (11.8–14.5)	13.4 (11.9–14.8)	13.5 (11.5–14.9)	13.1 (11.9–13.8)	13.5 (12.0–14.5)	13.0 (11.6–14.1)	0.290 ^A^
Previous abdominopelvic surgery, *n* (%)	32 (20.6%)	5 (16.1%)	6 (19.4%)	8 (25.8%)	6 (19.4%)	7 (22.6%)	0.906 ^P^
Neoadjuvant CCRT, *n* (%)	22 (14.2%)	6 (19.4%)	2 (6.5%)	2 (6.5%)	8 (25.8%)	4 (12.9%)	0.125 ^P^

SD: standard deviation; IQR: interquartile range; CCRT: Concurrent Chemo-radiotherapy. ^K^ Kruskal Wallis test. ^P^ chi-squared test. ^A^ ANOVA test.

**Table 3:** Tumor histopathological characteristics.

Parameters	Overall (*n* = 155)	Group 1 (*n* = 31)	Group 2 (*n* = 31)	Group 3 (*n* = 31)	Group4 (*n* = 31)	Group 5 (*n* = 31)	*p* Value
Tumor location							0.313 ^P^
Right-sided colon, *n* (%)	29 (18.7%)	5 (16.1%)	8 (25.8%)	9 (29.0%)	2 (6.5%)	5 (16.1%)	
Left-sided colon, *n* (%)	65 (41.9%)	14 (45.2%)	14 (45.2%)	11 (35.5%)	16 (51.6%)	10 (32.3%)	
Rectum, *n* (%)	61 (39.4%)	12 (38.7%)	9 (29.0%)	11 (35.5%)	13 (41.9%)	16 (51.6%)	
Malignant tumor, *n* (%)	136 (87.7%)	28 (90.3%)	25 (80.6%)	27 (87.1%)	29 (93.5%)	27 (87.1%)	0.620 ^P^
T4 lesion	16 (10.3%)	1 (3.2%)	4 (12.9%)	4 (12.9%)	3 (9.7%)	4 (12.9%)	0.668 ^P^
Stage							0.462 ^P^
* Stage 0	10 (6.5%)	3 (9.7%)	1 (3.2%)	2 (6.5%)	2 (6.5%)	2 (6.5%)	
Stage I	26 (16.8%)	6 (19.4%)	1 (3.2%)	5 (16.1%)	5 (16.1%)	9 (29.0%)	
Stage II	33 (21.3%)	9 (29.0%)	7 (22.6%)	8 (25.8%)	3 (9.7%)	6 (19.4%)	
Stage III	49 (31.6%)	5 (16.1%)	13 (41.9%)	10 (32.3%)	12 (38.7%)	9 (29.0%)	
Stage IV	9 (5.8%)	3 (9.7%)	2 (6.5%)	1 (3.2%)	2 (6.5%)	1 (3.2%)	
Median length of safe margin, cm (IQR)	2.4 (1.5–4.0)	2.3 (1.9–4.1)	4.0 (1.8–4.5)	2.2 (1.0–4.0)	2.8 (2.0–3.5)	2.0 (1.0–3.0)	0.173 ^K^
No residual tumor after neoadjuvant therapy	28 (18.1%)	5 (16.1%)	7 (22.6%)	5 (16.1%)	7 (22.6%)	4 (12.9%)	0.814 ^P^

* Stage 0 = pTisN0M0, ^K^ Kruskal–Wallis test, ^P^ chi-squared test.

**Table 4:** ERAS compliance rate.

Parameters	Overall (*n* = 155)	Group 1 (*n* = 31)	Group 2 (*n* = 31)	Group 3 (*n* = 31)	Group4 (*n* = 31)	Group 5 (*n* = 31)	*p* Value
Total ERAS protocol, % (Median, range)	83.3% (50.0–94.4)	72.2% (50.0–88.9)	83.3% (66.7–94.4)	83.3% (72.2–88.9)	83.3% (72.2–94.4)	83.3% (72.2–94.4)	<0.001 ^K^
Preadmission items	100% (50.0–100)	75.0% (75.0–100)	100% (50.0–100)	100% (50.0–100)	100% (50.0–100)	100% (50.0–100)	0.486 ^K^
Preoperative items	100% (75.0–100)	100% (75.0–100)	100% (75.0–100)	100% (75.0–100)	100% (75.0–100)	100% (75.0–100)	0.161 ^K^
Intraoperative items	100% (33.3–100)	66.7% (33.3–100)	66.7% (33.3–100)	100% (66.7–100)	100% (66.7–100)	100% (66.7–100)	<0.001 ^K^
Postoperative items	71.4% (14.3–100)	57.1% (14.3–85.7)	71.4% (28.6–100)	71.4% (28.6–100)	71.4% (42.9–100)	71.4% (42.9–100)	<0.001 ^K^

^K^ Kruskal–Wallis test.

**Table 5:** Perioperative data and clinical outcomes analysis of patient groups.

Parameters	Overall (*n* = 155)	Group 1 (*n* = 31)	Group 2 (*n* = 31)	Group 3 (*n* = 31)	Group 4 (*n* = 31)	Group 5 (*n* = 31)	*p* Value
Robotic method, *n* (%)	79 (51%)	0	13 (42%)	15 (48.4%)	23 (74.2%)	28 (90.3%)	<0.001 ^P^
Operative time, minutes							0.088 ^K^
Mean (SD)	250.3 (83.3)	229.3 (92.6)	242.7 (96.0)	258.1 (75.3)	262.7 (87.0)	258.9 (61.5)	
Median (IQR)	235 (195–297.5)	220 (182.5–232.5)	225 (169–285)	250 (210–310)	242 (195.5–315.5)	255 (207–282.5)	
Minimal blood loss, *n* (%)	129 (83.2%)	22 (71%)	25 (80.6%)	28 (90.3%)	25 (80.6%)	29 (93.5%)	0.130 ^P^
Stoma construction, *n* (%)	29 (18.7%)	5 (16.1%)	2 (6.5%)	5 (16.1%)	11 (35.5%)	6 (19.4%)	0.059 ^P^
Drainage tube placement, *n* (%)	73 (47.1%)	29 (93.5%)	28 (90.3%)	5 (16.1%)	5 (16.1%)	6 (19.4%)	<0.001 ^P^
Visual analog scale ≤ 3, *n* (%)	127 (81.9%)	25 (80.6%)	24 (77.4%)	23 (74.2%)	29 (93.5%)	26 (83.9%)	0.329 ^P^
First oral intake within 24 h, *n* (%)	137 (88.4%)	26 (83.9%)	21 (67.7%)	30 (96.8%)	30 (96.8%)	30 (96.8%)	0.001 ^P^
Mean time to first stool *, day ± SD	2.5 ± 1.8	3.9 ± 1.8	2.8 ± 2.0	2.1 ± 1.2	2.0 ± 2.0	1.6 ± 1.0	<0.001 ^K^
Mean IV amount, ml (SD)							
POD-0	1573.4 (810.3)	1145.4 (555.6)	1822.9 (738.4)	1658.1 (929.6)	1511.3 (707.1)	1729.3 (928.7)	0.005 ^K^
POD-1	1478.5 (728.3)	1533.4 (822.1)	1811.8 (1013.4)	1277.6 (638.6)	1312.3 (390.6)	1457.2 (520.3)	0.230 ^K^
POD-2	927.3 (904.8)	1198.1 (905.2)	1448.4 (1104.0)	551.6 (680.1)	782.1 (852.5)	656.5 (613.0)	0.001 ^K^
POD-3	644.3 (945.9)	1034.6 (935.7)	1461.7 (1045.5)	304.0 (640.4)	275.8 (738.6)	145.5 (551.0)	<0.001 ^K^
Mean urine amount, ml (SD)							
POD-1	1771.5 (884.6)	1714.8 (794.9)	2029.2 (1065.1)	1410.8 (764.1)	1662.7 (739.0)	2046.6 (912.7)	0.029 ^K^
POD-2	2019.9 (902.0)	2253.8 (1069.7)	2109.6 (958.7)	1621.0 (593.9)	2010.7 (852.3)	2115.4 (902.4)	0.139 ^K^
POD-3	2039.5 (879.6)	2178.6 (916.9)	2104.1 (894.5)	1837.9 (672.4)	1978.7 (711.4)	2139.7 (1232.0)	0.663 ^K^
** Optimal recovery, *n* (%)	85 (54.8%)	4 (12.9%)	11 (35.5%)	21 (67.7%)	24 (77.4%)	25 (80.6%)	<0.001 ^P^
^$^ Any complications, *n* (%)	24 (15.5%)	9 (29.0%)	7 (22.6%)	4 (12.9%)	1 (3.2%)	3 (9.7%)	0.078 ^P^
Major (grade 3–5)	8 (5.2%)	5 (16.1%)	2 (6.5%)	1 (3.2%)	0	0	
Minor (grade 1–2)	16 (10.3%)	4 (12.9%)	5 (16.1%)	3 (9.7%)	1 (3.2%)	3 (9.7%)	
Postoperative LOS							
Day, median (Q1–Q3)	5 (4–7)	8 (6–9)	7 (5–10)	5 (4–6)	4 (4–5)	4 (4–5)	<0.001 ^K^
More than 5 days, *n* (%)	67 (43.2%)	26 (83.9%)	20 (64.5%)	9 (29%)	7 (22.6%)	5 (16.1%)	<0.001 ^P^
Readmission within 30 days, *n* (%)	7 (4.5%)	3 (9.7%)	2 (6.5%)	1 (3.2%)	0	1 (3.2%)	0.421 ^P^

VAS: Visual Analogue Scale; * Patient with stoma construction were excluded. ** Optimal recovery was defined as absence of major complications, LOS no more than 5 days and no readmission within 30 days. ^$^ Complications were graded by Calvin–Dindo classification. ^K^ Kruskal–Wallis test, ^P^ chi-squared test.

**Table 6:** Comparison of ERAS compliance and LOS between laparoscopic and robotic surgery.

Parameters	Overall (N = 155)	Laparoscopic (N = 76)	Robotic (N = 79)	*p* Value
Total ERAS protocol, % (Median, range)	88.9 (55.6–100.0)	83.3 (55.6–94.4)	88.9 (72.2–100.0)	<0.001 ^m^
Preadmission items	100.0 (50.0–100.0)	87.5 (50.00–100.0)	100.0 (50.0–100.0)	0.14 ^m^
Preoperative items	100.0 (75.0–100.0)	100.0 (75.00–100.0)	100.0 (75.0–100.0)	0.100 ^m^
Intraoperative items	100.0 (33.3–100.0)	66.7 (33.33–100.0)	100.0 (33.3–100.0)	<0.001 ^m^
Postoperative items	85.7 (28.8–100.0)	71.4 (28.8–100.0)	85.7 (42.9–100.0)	<0.001 ^m^
Postoperative LOS				
Day, median (Q1–Q3)	5 (4–7)	6 (5–9)	4.1 (3.8–5.0)	0.016 ^m^
More than 5 days, *n* (%)	67 (43.2%)	48 (63.1%)	19 (24%)	<0.001 ^y^

^m^ Mann–Whitney U test, ^y^ Yates’ Correction for Continuity.

**Table 7:** Multivariable logistic regression model of factors associated with postoperative length of stay no more than 5 days.

	LOS ≤ 5d		
Factors	OR	95% CI	*p* Value
Robotic surgery	5.029	1.321 to 19.412	0.018
Quintile group sequence	1.270	0.779 to 2.070	0.337
Compliance of total ERAS protocol	0.995	0.918 to 1.079	0.910
Male	0.758	0.256 to 2.244	0.617
Age	0.996	0.958 to 1.036	0.849
BMI	1.087	0.961 to 1.229	0.184
ASA ≤ 2	4.560	1.262 to 16.382	0.020
Any comorbidity	0.816	0.288 to 2.311	0.702
Pre-op hemoglobin	1.198	0.930 to 1.542	0.163
Previous abdominopelvic surgery	0.803	0.265 to 2.435	0.698
Multiple primary cancers	0.430	0.036 to 5.185	0.507
Neoadjuvant CCRT	0.336	0.045 to 2.485	0.285
Cigarette use	1.773	0.458 to 6.862	0.407
Malignant disease	0.870	0.218 to 3.471	0.843
T4 lesions	1.205	0.243 to 5.981	0.819
Minimal blood loss	0.688	0.171 to 2.772	0.599
Stoma construction	1.721	0.261 to 11.356	0.573
Drainage tube placement	0.143	0.042 to 0.490	0.002
Operative time	0.989	0.980 to 0.998	0.019

## Data Availability

The data that support the findings of this study are available from the first or/and the corresponding author upon reasonable request.

## Supplementary Materials

The following supporting information can be downloaded at: https://www.mdpi.com/article/10.3390/jcm12072661/s1, Figure S1: Elements of tailored ERAS protocol and overall compliance rate; Table S1: Uni- and multivariate logistic regression analysis for ERAS compliance ≥ 75%.
