# Supplementary material for: Developing a Robotic Surgical Platform Is Beneficial to the Implementation of the ERAS Program for Colorectal Surgery: An Outcome and Learning Curve Analysis
Source: J Clin Med. 2023 Apr 3;12(7):2661. doi: 10.3390/jcm12072661 (PMC10095021; doi:10.3390/jcm12072661)
Supplement: Supplementary file 1 [file jcm-12-02661-s001.zip › jcm-2301420-supplementary.pdf]

Table S1. Uni- and multivariate logistic regression analysis for ERAS compliance ≥ 75%.

	Total Protocol		Intraoperative Items		Postoperative Items	
	**Univariate OR (95%CI, *p* Value)**	**Adjusted OR (95%CI, *p* Value)**	**Univariate OR (95%CI, *p* Value)**	**Adjusted OR (95%CI, *p* Value)**	**Univariate OR (95%CI, *p* Value)**	**Adjusted OR (95%CI, *p* Value)**
Robotic surgery	10.27 (2.27–46.40, 0.002)	1.81 (0.22–15.30, 0.585)	4.97 (2.51–9.82, <0.001)	1.04 (0.42–2.59, 0.934)	4.20 (2.14–8.25, <0.001)	1.83 (0.80–4.15, 0.151)
Quintile group sequence	8.17 (2.91–22.92, <0.001)	10.25 (2.52–41.64, 0.001)	3.41 (2.37–4.92, <0.001)	3.39 (2.25–5.10, <0.001)	1.97 (1.50–2.57, <0.001)	1.74 (1.27–2.38, 0.001)

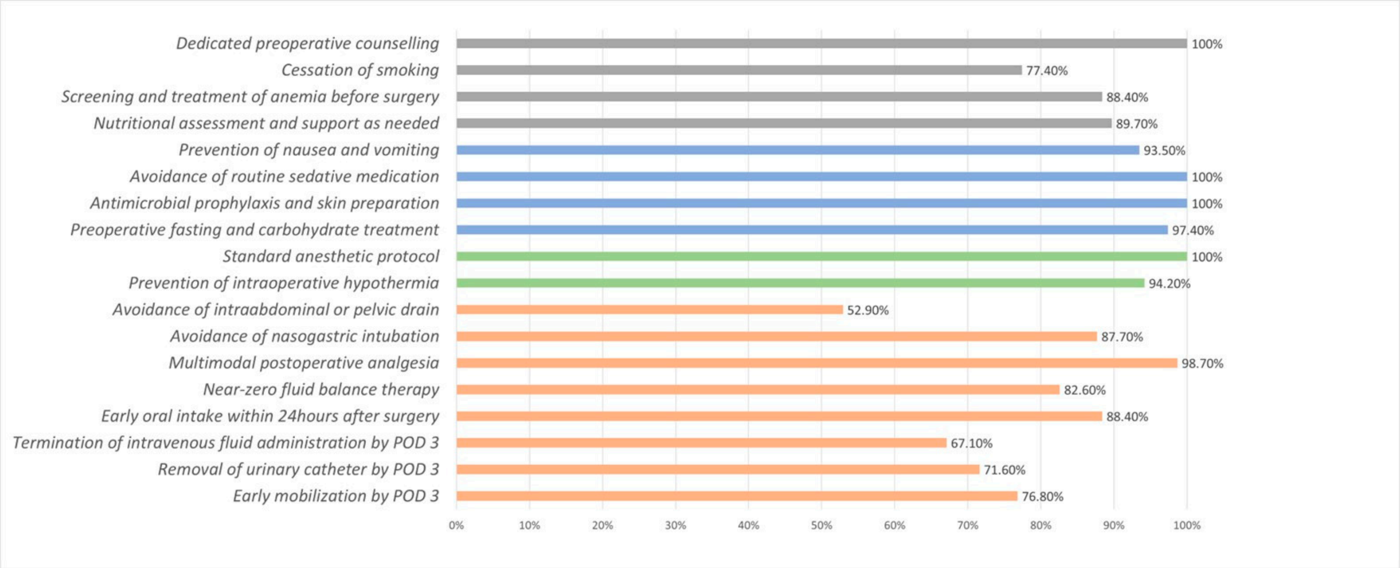

Figure S1. Elements of tailored ERAS protocol and overall compliance rate.
